# Hypertrophic Cardiomyopathy and the Brockenbrough-Braunwald-Morrow Phenomenon: A Case Report

**DOI:** 10.7759/cureus.5826

**Published:** 2019-10-02

**Authors:** Alexa Bello, Jose L. Diaz, Taylor P Travis, Joseph Varon, Salim R Surani

**Affiliations:** 1 Research, Dorrington Medical Associates, Houston, USA; 2 Pulmonary and Critical Care, Dorrington Medical Associates, Houston, USA; 3 Cardiology, Coastal Cardiology PLLC, Corpus Christi, USA; 4 Critical Care, United General Hospital, Houston, USA; 5 Internal Medicine, Texas A&M Health Science Center, Temple, USA

**Keywords:** hypertrophic cardiomyopathy, brockenbrough-braunwald-morrow phenomenon, septal myectomy, alcohol septal ablation

## Abstract

The Brockenbrough-Braunwald-Morrow phenomenon provides objective evidence of the existence and degree of left ventricular outflow tract (LVOT) obstruction, which can be improved with pharmacological therapy, surgical myectomy, or interventional alcohol septal ablation (ASA). This article incorporates contemporary research findings that are useful for the diagnosis and management of this entity.

We present the case of a 67-year-old lady with a past medical history significant for hypertension, hyperlipidemia, and coronary artery disease. The patient presented with a complaint of functional class-3 dyspnea on exertion with associated substernal chest tightness radiating to her back that had been worsening for two days prior to admission. An echocardiogram showed left ventricular hypertrophy with septal predominance measuring 17.5 mm in end-diastole and a left ventricular ejection fraction greater than 65%. The LVOT peak gradient was elevated and a positive Brockenbrough-Braunwald-Morrow phenomenon was observed for which a septal myectomy and coronary bypass of the left internal mammary artery (LIMA) to the left anterior descending (LAD) artery were performed. The patient had an uneventful postoperative course and her symptoms improved significantly.

The Brockenbrough-Braunwald-Morrow phenomenon is useful to determine the degree of LVOT and to confirm the resolution of obstruction after treatment.

## Introduction

Hypertrophic cardiomyopathy (HCM) is a genetic cardiac disease that is predominantly obstructive. This condition is characterized by hypertrophy of the interventricular septum in the absence of any other cardiac or systemic disease, and it is frequently associated with angina, syncope, and congestive heart failure, which are evident in about 70% of the patients [1]. This clinical entity has been studied for more than 50 years, and it has been also known to be the cause of arrhythmic sudden cardiac death, heart failure, and atrial fibrillation [2]. An evaluative hemodynamic maneuver known as the Brockenbrough-Braunwald-Morrow phenomenon, which is a classic haemodynamic trick that involves waveform interpretation as well as invasive measurement, can provide some evidence of the existence and degree of left ventricular outflow tract (LVOT) obstruction [3]. In patients with no evidence of resting obstruction, the Brockenbrough-Braunwald-Morrow phenomenon is a helpful maneuver to document the degree of dynamic LVOT obstruction in perioperative alcohol septal ablation (ASA) as well as prior to myectomy, and it can confirm the resolution of obstruction after the intervention [3]. The treatment includes pharmacological therapy for which beta-blockers remain the gold standard to improve symptoms. However, some patients have refractory symptoms and significant obstruction. For these carefully selected patients, interventional techniques such as septal myectomy or ASA may reduce the outflow tract gradient more effectively [4]. In this study, we present a case of hypertrophic cardiomyopathy with the presence of Brockenbrough sign.

## Case presentation

A 67-year-old woman with a past medical history significant for hypertension, hyperlipidemia, and coronary disease presented to the hospital complaining of functional class-3 dyspnea on exertion with associated substernal chest tightness. The chest tightness radiated to her back and had been worsening for two days prior to admission. 

The patient denied taking any nitroglycerin and admitted to having taken an extra dose of metoprolol. She denied orthopnea or paroxysmal nocturnal dyspnea. The patient had past coronary disease treated with a prior percutaneous coronary intervention (PCI) of her mid-left anterior descending artery. Due to the progressive dyspnea on exertion concerning for unstable angina, she was taken for left-heart catheterization.

Vital signs revealed a blood pressure of 96/55 mmHg, heart rate of 51 beats per minute, respiratory rate of 26 breaths per minute, temperature of 98.7ºF, and oxygen saturation of 99% while breathing on room air. On cardiac auscultation, she had bradycardia with a regular rhythm. S1 and S2 sounds were normal with a systolic flow murmur of grade III/VI. Blood chemistry showed chloride of 108 mEq/L (normal range: 95-105 mEq/L), potassium of 4.9 mEq/L (normal range: 3.5-5 mEq/L), and sodium of 123 mEq/L (normal range: 136-145 mEq/L). The complete blood count was normal. An electrocardiogram (EKG) showed normal sinus rhythm with down-sloping ST depressions and T-wave inversions. Echocardiographic findings revealed left ventricular hypertrophy with septal predominance measuring 17.5 mm in end-diastole and a left ventricular ejection fraction greater than 65%. The LVOT peak gradient was elevated at 175 mmHg, and her left atrium was mildly dilated with an index of 32 mL/m2.

Cardiac enzymes showed a creatine kinase muscle/brain (CK-MB) of 9.5 ng/mL (normal range: 5-25 IU/L), and initial Troponin I of 0.105 ng/mL (normal range: 0-0.5 ng/mL). Troponin I fluctuated to different values within the first 24 hours of admission. It went from 0.105 ng/mL to 0.089 ng/mL in the first couple of hours, then decreased to 0.065 ng/mL and finally went down to 0.053 ng/mL within 24 hours.

**Figure 1:**
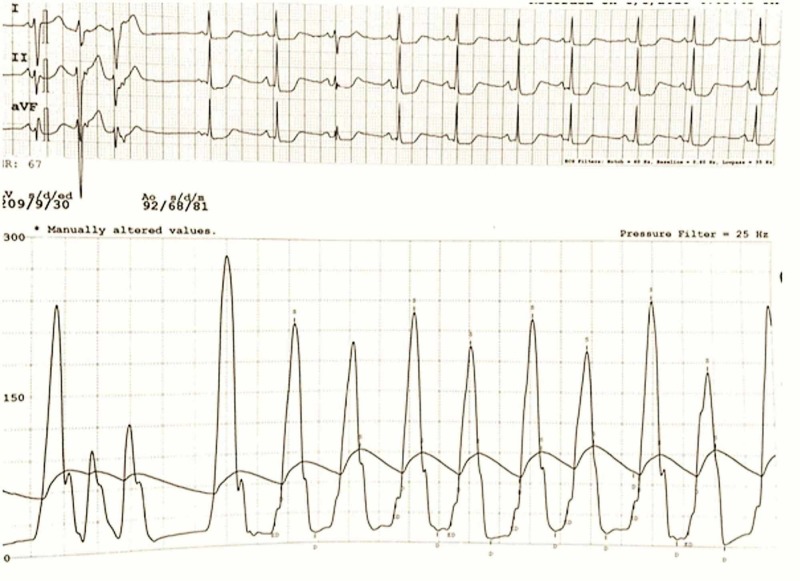
Tracing of the Brockenbrough-Braunwald-Morrow sign. The aortic waveform is often dampened in real life due to the small luminal area of the aortic portion of the Langston dual lumen catheter

Cardiac catheterization revealed 50% stenosis in the ostial left anterior ascending artery and 60% stenosis in the third obtuse marginal. During the intervention, a dual lumen pigtail catheter was inserted across the aortic valve with the distal lumen entering the left ventricle and the proximal lumen remaining in the aorta. Left ventricular pressure recording suggested HCM with an increased left ventricular to the aortic gradient of 210 mmHg after premature ventricular contractions (PVCs), and a positive Brockenbrough-Braunwald-Morrow phenomenon (Figure 1). The patient was referred for surgery and a septal myectomy and LIMA to the LAD coronary bypass were performed. The patient had an uneventful postoperative course and currently has functional class-one symptoms on her outpatient follow-up.

## Discussion

HCM remains an important cause of disability and death in patients of all ages [5]. It affects both genders and has a prevalence of one in every 500 people, with similar causal mutations, clinical course, and phenotypic expression despite their racial or ethnic origins [2,5]. Epidemiological data over the past few years have shown that in Europe, Japan, China, East Africa, and even in the US, this entity has a prevalence of one in 500 [2]. This clinical condition has a complex, autosomal dominant pattern of inheritance that possesses a 50% chance of phenotypic expression [2]. HCM has been found to be caused by a variety of dominant mutations in at least 11, and up to 20, or more genes that are in charge of encoding proteins of the cardiac sarcomere or the Z-disc, its components, and functions [2,6]. It has been documented that 70% of the cases are associated with mutations in the two main genes: beta-myosin heavy chain (MYH7) and the myosin-binding protein C (MYBPC) [2,5-7]. These two genes are the major components of the sarcomere, which provides structural support as well as regulate muscle contraction [6]. Recent studies have shown that mutations in the troponin T (TNNT2) gene among several other genes such as the α-myosin heavy chain (MYH6), titin (TTN), muscle LIM protein (CSRP3), telethonin (TCAP), vinculin (VCL), and junctophilin 2 (JPH2) are linked to this entity as well. However, these genes represent only 3-5% of the cases of HCM [2].

This phenomenon has been the subject of intense investigation for a long time due to its important role in causing disability and death in patients of all ages, although sudden and unexpected death in young people including trained athletes is the worst component of its natural history [5]. HCM is a predominantly obstructive disease characterized by hypertrophy of the interventricular septum (IVS) and systolic anterior motion (SAM) of the mitral valve resulting in LVOT obstruction. If SAM is not present, a mid-ventricular obstruction or abnormalities of the mitral valve are found. The aim of the treatment is to reduce SAM to eliminate the intracavitary pressure gradient (PG) in order to improve myocardial performance and symptoms [4].

The Brockenbrough-Braunwald-Morrow phenomenon has been demonstrated when a paradoxical decline in arterial pulse pressure occurs after an ectopic beat, usually a premature ventricular contraction. This sign was first described by Brockenbrough et al. in 1961 [1,3,8]. After a PVC, there is a compensatory pause that causes an increase in the diastolic filling time and subsequently expands the diastolic volume. Following the Frank-Starling mechanisms, the stroke volume increases as a result of an increase in end-diastolic volume when this remains constant, resulting in an increase of cardiac muscle stretch leading to an increase in myocardial contractility causing a rising of the arterial pulse pressure [9,10].

The clinical diagnosis can be confirmed by imaging with a transthoracic echocardiogram or cardiac MRI. The most common findings include left ventricular wall thickness (21-22 mm), which is also associated with mild right-ventricular hypertrophy. In some other cases, mitral valve SAM is present as well as a hyperdynamic left ventricle, which are not obligatory but can be present in the diagnosis of HCM [2]. In patients with hypertrophic obstructive cardiomyopathy (HOCM), the increased preload after a PVC worsens the LVOT obstruction, causing a decrease in the arterial pulse pressure and the Brockenbrough-Braunwald-Morrow sign [9]. 

In our patient, the echocardiogram showed left ventricular hypertrophy with septal predominance measuring 17.5 mm in end-diastole and a left ventricular ejection fraction greater than 65%. The LVOT peak gradient was elevated at 175 mmHg and her left atrium was mildly dilated with an index of 32 mL/m2. In addition, the left ventricular pressure recording was compatible with HCM with an increased left ventricular to the aortic gradient of 210 mmHg after PVCs, and a positive Brockenbrough-Braunwald-Morrow phenomenon was observed.

The Brockenbrough-Braunwald-Morrow phenomenon can be useful in the cardiac catheterization laboratory for those patients with substantial symptoms and increased exertional gradient not seen at rest. Up to 75% of patients with HOCM have no documented gradient at rest. Thus, the tactic can be employed to document the degree of dynamic LVOT obstruction in perioperative ASA as well as prior to myectomy, and it can confirm the resolution of obstruction after the intervention. Therefore, it assesses the adequacy of septal reduction after surgical myectomy or ASA [2,3]. ASA has been indicated for patients with symptomatic, medically refractory HCM [3]. 

ASA has been shown to have similar outcomes when compared to septal myectomy, which is currently thought of as the first-line treatment for managing symptomatic patients with HOCM [9,11]. It was first performed in 1995 and has been demonstrated to be a hemodynamically effective method of reducing LVOT obstruction [12]. This technique is a percutaneous alternative to surgical septal myectomy. It creates a septal infarction and has an intracoronary approach that utilizes ethanol injection to the septal perforator branch, which induces a localized infarction of the basal septum at the site of contact of the anterior mitral valve leaflet so that the LV outflow tract gradient is reduced [11]. However, a disadvantage of this technique is the presence of an increased risk of ventricular arrhythmias as a result of the ischemia present in the early post-procedural state [7]. This procedure is indicated for patients who remain symptomatic with gradients of >30 mmHg or evidence of exercise gradients of >60 mmHg despite optimal medical therapy [9]. However, young patients and those with severe, diffuse, or thin septal thickness with a very high LVOT gradient are found to be poor candidates [11]. On the other hand, septal myectomy, which is also known as the Morrow procedure, has been considered the gold standard of invasive therapies for severely symptomatic HCM patients. This surgical technique has been established as the most effective and proven approach for reversing the consequences of heart failure by providing amelioration of obstruction (and relief of mitral regurgitation) at rest, with the restoration of functional capacity and acceptable quality of life at any age [13]. The transaortic method remains the primary method of exposure, in which an extended muscular resection is performed [2,7]. This procedure consists of resecting a small amount of muscle (5 g) from the proximal septum and beyond the distal margins of the mitral leaflets without producing intra-myocardial scarring [2].

In higher volumes and highly experienced centers, septal myectomy has been shown to have less than one percent mortality and a 70% improvement in symptoms and exercise capacity of five years or more after the operation [2,14]. Luke and associates showed that low septal myectomy volume has been associated with increased mortality, a longer length of stay and higher costs. Therefore, the referral of patients to highly experienced centers for both procedures is suggested for better outcomes [14]. In our patient, a septal myectomy and LIMA to the LAD coronary bypass were performed with an uneventful postoperative course. However, both ASA and septal myectomy can result in the reduction of the LVOT gradient, improvement in the heart-failure symptoms, as well as obstruction relief. Evidence supports better long-term symptom relief in patients who undergo septal myectomy [11]. Choosing the best interventional treatment in HCM patients with dynamic LVOT obstruction relies on the following factors: demographics such as the age of the patient, presence of comorbidities, and patient’s choice. Anatomic, electrical, and hemodynamic criteria are also taken into consideration [7].

## Conclusions

HCM is a clinical condition that needs to be addressed properly in order to prevent severe complications and sudden death in patients. Although many patients improve with pharmacological therapy, interventional options such as septal myectomy and ASA should be considered for some patients. As shown in our case, our patient underwent septal myectomy with an uneventful course with an improvement of the symptoms. Both ASA and septal myectomy improve symptoms and reduce gradients. Septal myectomy is considered the gold standard to treat a patient with HCM. However, ASA should also be considered as an alternative as it has been shown to provide results in the treatment of this pathology.
